# Cancer survivors in Switzerland: a rapidly growing population to care for

**DOI:** 10.1186/1471-2407-13-287

**Published:** 2013-06-14

**Authors:** Christian Herrmann, Thomas Cerny, Anita Savidan, Penelope Vounatsou, Isabelle Konzelmann, Christine Bouchardy, Harald Frick, Silvia Ess

**Affiliations:** 1 Cancer Registry St. Gallen-Appenzell, Cancer League Ostschweiz, St Gallen, Switzerland; 2 Department Epidemiology and Public Health, Swiss Tropical and Public Health Institute, Basel, Switzerland; 3 University of Basel, Basel, Switzerland; 4 Medical Oncology-Hematology Department, Kantonsspital St. Gallen, St. Gallen, Switzerland; 5 Valais Cancer Registry, Sion, Switzerland; 6 Geneva Cancer Registry, Geneva Faculty of medicine, Geneva, Switzerland; 7 Cancer Registry Graubünden-Glarus, Chur, Switzerland

**Keywords:** Cancer survivors, Survivorship, Survivorship care, Complete prevalence, Time trends, Projections, Switzerland

## Abstract

**Background:**

Cancer survivors are a heterogeneous group with complex health problems. Data concerning its total number and growing dynamics for Switzerland are scarce and outdated.

**Methods:**

Population and mortality data were retrieved from the Swiss Federal Statistical Office (FSO). Incidence and relative survival for invasive cancers were computed using data from the cancer registries Geneva (1970–2009), St. Gallen - Appenzell (1980–2010), Grisons & Glarus (1989–2010), and Valais (1989–2010). We estimated prevalence for 1990–2010 using the Prevalence, Incidence Approach MODel (PIAMOD) method. We calculated trends in prevalence estimates by Joinpoint analysis. Projections were extrapolated using the above models and based on time trends of the period 2007–2010.

**Results:**

The estimated number of cancer survivors increased from 139′717 in 1990 (2.08% of the population) to 289′797 persons in 2010 (3.70%). The growth rate shows an exponential shape and was 3.3% per year in the period 2008 to 2010. Almost half of the survivors have a history of breast, prostate or colorectal cancer. Among cancer survivors, 55% are women but the increases have been more marked in men (p < 0.01, 3.9% annual increase in men vs. 2.7% in women since 2008). By the end of 2020 372′000 cancer survivors are expected to live in Switzerland.

**Conclusions:**

There is a rapidly growing population of cancer survivors in Switzerland whose needs and concerns are largely unknown.

## Background

As a consequence of improved life expectancy, of the growth and the aging of the population, as well as cancer awareness and early detection strategies the number of new cancer cases has raised continuously in the last 30 years in Switzerland and worldwide [1]. At the same time, due to earlier diagnosis and improved treatments, cancer mortality has declined and survival rates have improved [2,3]. The conjunction of these factors has led to a large and rapidly growing number of cancer survivors.

There are several definitions of cancer survivor. Here, we use the term of cancer survivor to describe any person alive with a previous diagnosis of cancer, following the American Society of Clinical Oncology (ASCO) [4] and others [5-7] who define survivorship as the “process of living with, through and beyond cancer”, equalling the definition of complete prevalence.

It is not until recently that the special needs of this growing population of cancer survivors have been brought into the focus of researchers and stakeholders. In 2006 the Institute of Medicine (IOM) focused on the transition from primary treatment to follow up care and the necessity to provide patients with a comprehensive care summary and follow-up plan for guidance on follow-up care, prevention and health maintenance [8].

In 2010 a National Cancer Survivorship Initiative in the UK recognized that not enough attention has been given to the long-term consequences of a cancer diagnosis and treatment and that action is needed in order to support cancer survivors to live as healthy and active a life as possible [9,10]. The ongoing needs of cancer survivors in Switzerland have received insufficient attention up to now.

Three distinct phases of cancer survival has been proposed: the first includes the time from diagnosis to the end of the initial treatment which may extend from some months to several years, the second includes the transition from treatment to extended survival and the third represents the long-term survival [11].

Cancer survivors will have greater health needs than the general population because the disease and/or treatment may lead to long-term or permanent impairment. Moreover, people with a history of cancer have an elevated risk for new primary cancers than the general population [12].

An increase in cancer survivors is expected to result in a need for additional specialized health personnel [8], and a substantial increase in training in survivorship care to support the delivery of multidimensional primary care for long-term survivors [13]. In a review from 2011, Richardson et al. [7] identified growing concern that the services required to meet the physical, social and emotional needs of survivors have not been adequately developed so far.

In order to adequately develop strategies and services required to meet the needs of this growing population updated epidemiological data is essential. In Switzerland, data on the number, growing dynamics and characteristics of cancer survivors are not available or outdated. Last published data for Switzerland correspond to prevalence estimates for 1992 and only for a limited number of malignancies [14].

The aim of the present work is to provide estimates of the number and characteristics of cancer survivors by the end of 2010 and project trends until 2020 in order to better understand the challenges that this booming population poses to oncological and general health services in the near future.

## Methods

### Data sources

To estimate the Swiss complete prevalence for the period 1990–2010, we used data provided by the registries Geneva (1970–2009), St. Gallen - Appenzell (1980–2010), Grisons-Glarus (1989–2010) and Valais (1989–2010). These registries, that cover approximately 26% of the Swiss population, are the only Swiss registries to satisfy following conditions i) have published incidence data in Cancer in 5 Continents Volume IX, ii) have incidence data at least from 1990 onwards and iii) are able to provide survival data. These data is routinely collected by the registries as part of national and cantonal programs. Following federal regulations, after anonymization excluding any identifiable information such as names and exact dates these data can be used in epidemiological studies without additional ethics committee approval.

Persons presenting with invasive cancers (International Classification of Disease, 10th edition, codes C0-C96, D45-D47) except non melanoma skin cancers [C44]) were included in the study. Individuals with multiple primaries were counted only once and considered to be prevalent since the first diagnosis of invasive cancer retrieved from the cantonal cancer registries. Aggregated population and mortality data for the corresponding cantons and for Switzerland by year, gender and age were retrieved from the Swiss Federal Statistical Office (FSO) [15].

Incident DCO (“death certificate only”) cases were excluded, as the true incidence date is unknown. The DCO rate was similar for all regions and varied in 1990–2010 in the different regions from 0.1-2.3% with an overall average of 0.6%. Cancer patients lost to follow-up were included and account for 3.8% (95% Confidence Interval, CI: 3.6%-4.1%) of studied population. This proportion declined during the study period and was 0.2% in 2010.

### Statistical methods

Complete cancer prevalence in Switzerland was estimated in a 3-step process by gender and cancer site, with all cancer sites being modelled as a single site. First, we estimated yearly incidence counts for Switzerland by single years of age using the pooled yearly incidence rates by age of the aforementioned registries and the population data of the FSO. Then, we estimated survival in Switzerland as the relative survival in the pooled cantons, where patients lost to follow-up were censored at time of last contact. And finally, with these data plus population and all-cause mortality data for Switzerland, we modelled cancer prevalence using the Prevalence, Incidence Approach MODel (PIAMOD) method [16]. The same software was used to project prevalence until 2020 basing the incidence estimation on the Age-Period-Cohort model with a linear period drift based on the period 2007 to 2010 and pertaining age and cohort effect. The survival, number of newborns and mortality for all competing causes are assumed to remain constant at the level of 2010. The absolute number of prevalent cases in 2020 was estimated by multiplying the projected prevalence rates for 2020 by the population count forecast of the FSO using their reference scenario (scenario no. A-00-2010) [17].

The tabulated relative survival in 6 month intervals for maximally 20 years of follow-up was calculated on the pooled dataset comparing observed survival with expected survival in Switzerland using the Ederer II method [18] with the so called mixed-approach [19] by consecutive 3-year periods from 1981 to 2010 and 5 distinct age-groups (0–14, 15–49, 50–69, 70–79, 80+). In the age group of 80+ years olds survival was restricted to a follow up duration of 15 years due to high variance in the survival estimates resulting from small number of cases.

The fit of the Age-Period-Cohort-model based incidence and hence prevalence was first evaluated on observed pooled incidence rates, and in a second step the final model parameters were selected by maximizing representativeness of the local data. Representativeness was measured by the sum of squared differences of the modelled expected mortality rates from observed national mortality rates. The observed national mortality rates were obtained from FSO data following the incidence selection criteria and using the applicable correction factors before the year 1995 because of the change of the directive of mortality codification occurred in our country [20]. For the final models the expected mortality differed per year averagely 4% for women and 5% for men in 1981–2010 from the national rates.

Temporal trends, their statistical significance and time points with significant changes in trend were assessed with Joinpoint models [21], using the JoinPoint Regression program of the National Cancer Institute. Joinpoint models were restricted to maximally 4 joinpoints and with a Poisson model of variation. A Monte Carlo Permutation method was used to test for a statistically significant change in trends. In addition, the goodness of fit of models with identity link (piecewise linear models) or log-link (for calculating annual percentage increases) was compared.

Aging trends in the population for 3 age groups (0–19, 20–64, 65–99) were analysed with Joinpoint models of the same kind.

## Results

**Figure 1:**
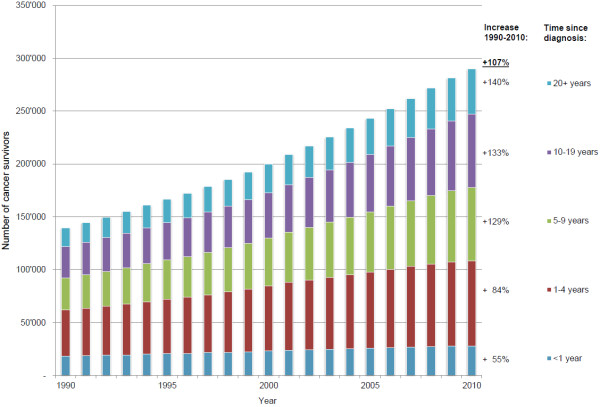
**Estimated number of cancer survivors in Switzerland by time since diagnosis.** For any invasive malignancy excluding non-melanoma skin cancers. Percentages denote increases in the period 1990–2010.

Figure 1 shows the exponential increase in the estimated number of those living with a history of cancer in Switzerland between the years 1990 and 2010 by time since diagnosis. Cancer survivors diagnosed less than 5 years ago constituted the largest group while the biggest rise is observed among very long term (20 years and more) survivors.

**Figure 2:**
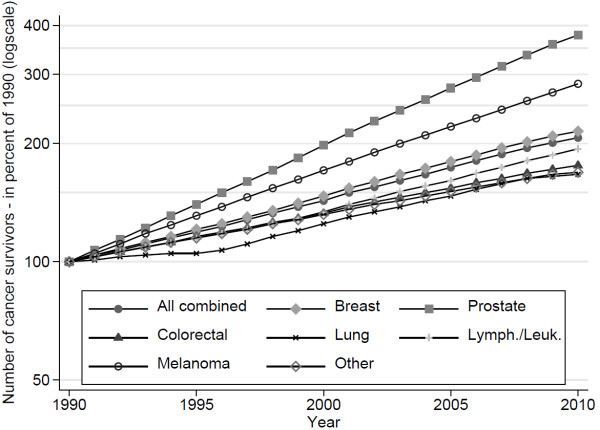
**Trend of prevalence of cancer survivors by type of cancer in Switzerland.** Expressed as the percent of 1990 value by type of cancer with highest incidence. Excluding non-invasive and non-melanoma skin cancers.

Table 1 and Figure 2 show time trends by type of cancer, gender, time since diagnosis and age group. The highest increase of cancer survivors prevalence was observed for prostate cancer with an almost 4 fold increase (+279% in 2010 vs. 1990), followed by skin melanoma (+184%) and breast cancer (+115%), all cancers with a high increase in incidence in the period studied. The estimate number of all cancer survivors was approximately 290′000 for the year 2010. For prevalence rates, the same pattern was seen.

**Table 1:** Trend in cancer prevalence in term of number of cases and crude rates per 100′000 in Switzerland between 1990 and 2010

	**Prevalence**							
	**Number of persons**				**Rates per 100'000**			
	**1990**	**2010**	**Increase**	[95%CI*]	**1990**	**2010**	**Increase**	[95%CI*]
**Gender**								
Men	56′201	132′330	+136%	[132%-141%]	1′714.5	3′436.2	+101%	[ 94%-108%]
Women	83′516	157′467	+89%	[ 85%- 92%]	2′431.8	3′962.6	+63%	[ 60%- 66%]
Total	139′717	289′797	+108%	[105%-111%]	2′081.5	3′703.5	+78%	[ 74%- 83%]
**Time since diagnosis**								
< 1 year	18′113	28′029	+55%	[ 51%- 60%]	269.8	417.6	+55%	[ 51%- 60%]
1-4 years	43′759	80′340	+84%	[ 76%- 93%]	651.9	1′196.9	+84%	[ 76%- 93%]
5-9 years	30′235	69′368	+129%	[118%-141%]	450.4	1′033.5	+129%	[118%-140%]
10-19 years	29′844	69′481	+133%	[123%-143%]	444.6	1′035.1	+133%	[123%-143%]
20+ years	17′765	42′578	+140%	[135%-145%]	264.7	634.3	+140%	[134%-145%]
Total	139′717	289′797	+108%	[105%-111%]	2′081.5	3′703.5	+78%	[ 74%- 83%]
Cancer type**								
Breast	30′892	66′513	+115%	[111%-119%]	460.2	850.0	+85%	[ 78%- 92%]
Prostate	12′012	45′421	+279%	[267%-291%]	179.0	580.5	+225%	[212%-239%]
Colorectal	16′186	28′567	+77%	[ 74%- 79%]	241.1	365.1	+52%	[ 49%- 55%]
Lung	4′689	7′833	+67%	[ 55%- 79%]	69.9	100.1	+44%	[ 36%- 53%]
Lymph./Leukaemia	13′470	26′086	+94%	[ 92%- 96%]	200.7	333.4	+67%	[ 57%- 78%]
Melanoma	8′367	23′743	+184%	[177%-191%]	124.6	303.4	+143%	[140%-147%]
Other	54′102	91′634	+70%	[ 66%- 73%]	806.0	1′171.1	+46%	[ 40%- 51%]
Total	139′717	289′797	+108%	[105%-111%]	2′081.5	3′703.5	+78%	[ 74%- 83%]
**Age group**								
0-14	740	1′061	+44%	[ 32%- 57%]	64.5	89.7	+40%	[ 31%- 49%]
15-49	21′192	36′285	+72%	[ 59%- 86%]	605.3	951.1	+57%	[ 56%- 59%]
50-69	53′236	112′657	+112%	[110%-114%]	3′848.7	5′949.8	+54%	[ 51%- 58%]
70-79	37′565	77′033	+106%	[ 95%-118%]	8′665.2	13′823.2	+60%	[ 54%- 67%]
80+	26′984	62′761	+132%	[112%-154%]	10′925.9	16′691.0	+53%	[ 47%- 60%]
Total	139′717	289′797	+108%	[105%-111%]	2′081.5	3′703.5	+78%	[ 74%- 83%]

*CI Confidence Interval.

**Excluding non-invasive and non-melanoma skin cancers.

In the same lapse of time the population grew by 12% (95% CI: 10%-15%) but the growth was unevenly among age groups: while the age group 65 years old and older grew by 36% (95% CI: 33%-38%) the number of those aged 0–19 increased only by 4% (95% CI: 1%-9%). Cancer incidence increased both in absolute numbers from estimated 24′335 in 1990 to 32′875 in 2010 (p < 0.01) and in relative numbers from a rate of 362.5 /100′000 in 1990 to 432.5/100′000 in 2010 (p < 0.01),while mortality decreased from 226.9/100′000 in 1990 to 209.9/ 100′000 in 2010 (p < 0.01). At the same time 10-year observed survival increased by 24% (95% CI: 21%-28%) from 0.27 to 0.34.

The increase was assessed to be exponential in comparing models assuming either piecewise linear or piecewise exponential increase, with the latter having better fit. The exponential increase, measured as annual percent change (APC), was at all periods significantly different from zero and higher in men than in women. In both genders the APC values increased from 1990 until a period around the year 2000 and declined since. For both genders combined the most recent (2008–2010) APC in the number of cancer survivors was 3.3% (95% CI: 3.1%-3.5%).

The results of our projection model showed a further continuation of this exponential increase for the next 10 years with an APC value close to the most recent one. We have estimated that until 2020 the total number of cancer survivors will increase by 28% to a total of 372′000 (i.e. 4.4% of the Swiss population). The projected number of cancer survivors and their increase since 2010 using the projection model by major cancer sites and gender can be found in Additional file 1. The biggest increases were predicted for melanoma and prostate cancer, while female breast cancer survivors were predicted to still be the biggest group.

## Discussion

In Switzerland, the overall number of cancer survivors has increased exponentially in the last 20 years and is expected to rise by about 30% in the next 10 years. We estimated that in 2010, 3.7% of the Swiss population were living with a history of cancer. This trend is the result of several factors i) the continuing advances in the treatment of oncologic diseases, ii) the spread of early detection of common types of cancer such as prostate, breast cancer and melanoma and iii) demographic changes: a growing segment of the aged population and an increased life expectancy due to various reasons. In particular, advances in treatment of cardiovascular diseases lead to a significant reduction of premature deaths [22].

Similar results both concerning trend and proportion of the population with a history of cancer have been reported in other European countries. In the Nordic countries (Sweden, Finland, Denmark, Norway, Iceland) 3.4 to 4.1% of the population is estimated to be a cancer survivor by the end of 2010 [23]. In the UK new estimations suggest that 2 million people representing 3.1% of the population live with a diagnosis of cancer in 2010 [24]. In Italy the projections for 2010 estimate that 4% of women and 3% of men are cancer survivors [25]. Similar pattern has also been reported in the USA [26] with estimated 13.7 millions of Americans alive with a history of cancer on January 1, 2012.

In these publications a wide range of methods to estimate cancer prevalence was used based on the availability of data in time and space and the underlying question.

The method we used was also applied in De Angelis et al. [25] and is specifically designed to estimate cancer prevalence in settings with incomplete registration, in contrast to e.g. discrete time models [24] where a long time series of cancer registry data is necessary. Additionally, our approach allowed the investigation of time trends and -in using Joinpoint regression- the assessment of significant changes therein over time.

The exponential increase in the past 20 years is mainly attributable to cancer incidence growth driven by screening uptake especially of prostate cancer and breast cancer and to a lesser extent to the aging of the population. PSA screening has lead in Switzerland and worldwide [27] to 3-4- fold increases of incidence rates of prostate cancer. Moreover, median age at diagnosis decreased, further contributing to increases of survival and prevalence of prostate cancer survivors. The incidence rate of breast cancer has also doubled although the reasons for this increase remain controversial [28,29]. At the same time considerable advances in treatments and supportive care have been realised in breast cancer and other types of cancers. In particular, the number of survivors with haematological malignancies and lung cancer that has increased by 94% and 67% respectively testifies of these advances.

It is not possible to predict with accuracy the total number of survivors in the future. In order to reflect the present situation we used for the projections the most recent trends (e.g. those in period 2007–2010). Future numbers will depend on the evolution of incidence, survival and demographic changes, and therefore might differ from current predicitons. E.g. incidence of breast cancer will probably increase as a consequence of the very recent introduction of mammography screening programs in many of the German-speaking cantons.

Most cancer survivors living with a cancer diagnosis since more than one and less than 5 years are in the phase following initial treatment, some of them disease free, others are under long term maintenance therapy managing sequelae of their treatment. Most of the cancer survivors in this phase will require additional treatment or special surveillance for relapse. Symptoms and problems may differ according to the type of cancer and the type of treatment applied. Breast cancer survivors may experience lymphedema of the arm, a common side effect of breast cancer surgery and radiation therapy that can develop soon after treatment or years later. Risk of lymphedema is reduced when sentinel node-biopsy rather than axillary dissection is performed to determine if the tumour has spread. In Switzerland less than 50% of women qualifying for sentinel node biopsy were operated with this technique in the years 2003 to 2005 [30]. Prostate cancer survivors treated with surgery or radiation therapy for early disease may experience symptoms and side effects of treatment including incontinence, erectile dysfunction and bowel complications [31]. Long term survivors of colorectal cancer may experience bowel problems and distress regarding cancer, specially fear of recurrence [32].

We have observed the biggest increases in number of survivors among long-term survivors. Because of increased life expectancy and improvement in therapies it is anticipated that this group will grow substantially in the coming decade. Late toxicities of therapies such as cardiotoxicity after cytotoxic drugs, cognitive deficits, osteoporosis etc., will develop in long-term survivors. Moreover, this group is at enhanced risk to other primary cancers and may suffer of poorer health. Several studies [33,34] have found that compared with individuals without a history of cancer or other chronic disease a substantial number of individuals who have a history of cancer were significantly more likely to report poor health and well-being, have a psychological disability, have limitations of activities of daily living and among those under the age of 65, being unable to work because of a health condition. These findings suggest the necessity of developing specific support for cancer survivors.

According to our estimates approximately one third of cancer survivors are younger than 65 and survivors in this age group have more than doubled in the 20 past years. In this age group resuming as normal a life as possible includes psychological and/or social support as well as professional reinsertion.

Cancer is increasingly an illness which might be cured or which might have the characteristics of a long term or chronic condition even in patients with advanced disease. It seems therefore essential to inform patients and future providers of the long-term effects of cancer and its treatment and to identify psychosocial needs and resources to guide prevention and health maintenance in order to increase the quality of life.

A major limitation of this study is the fact that we based our estimation on the data from only 4 regional cancer registers covering about 26% of the population. As in the USA, where complete prevalence estimates are based only on SEER registries [35], it is only in 2012 that the coverage of cancer registration attained 80% in Switzerland. However the data used are of high quality as ascertained by the controls performed at the International Agency for Research on Cancer [1], with high levels of microscopic verification (95%) and very low level of DCO cases (0.6%). Furthermore the data cover rural alpine and urban areas in the main language regions (French speaking and German speaking regions) and include administrative units (cantons) with and without breast cancer screening programs. Additionally, the model parameters were chosen by minimizing squared differences of modelled with observed national mortality rates, and thus maximizing representativeness of the available data. The expected mortality rates of the final models showed very close fit to the national mortality rates. We are therefore confident that estimates reflect the true situation in Switzerland as close as possible.

This study is a first step into understanding the number and characteristics of people living with a cancer diagnosis in Switzerland. More research is needed to know the health status, the quality of life and the expectation of cancer survivors, their need in care and support. This will enable resource planners to better translate the available information into necessary formation of professionals, health care and social structures to adequately meet the specific needs of cancer survivors.

## Conclusions

The success of cancer research, early diagnosis and treatment over the last 20 years as well as increases in life expectancy have led to exponential increases of individuals living many years with a cancer diagnosis. Further research is needed to better understand the special needs of survivors and to implement care according to these needs.

## Competing interests

The authors declare that they have no competing interests.

## Authors’ contributions

TC, SE conceived of the study. CH carried out the analysis. CH, SE, TC contributed to the interpretation of the data and the writing of the manuscript. IK, CB, HF and SE contributed to the data acquisition. TC, AS, PV IK, CB, HF and SE critically revised the manuscript. All authors read and approved the final manuscript.

## Pre-publication history

The pre-publication history for this paper can be accessed here:

http://www.biomedcentral.com/1471-2407/13/287/prepub

## Supplementary Material

Additional file 1 **Projected cancer prevalence in Switzerland for 2020.** This table presents the projected cancer prevalence in terms of number of cases by gender for major cancer sites in Switzerland for the year 2020. Click here for file
